# High-Throughput Assessment of Real-World Medication Effects on QT Interval Prolongation: Observational Study

**DOI:** 10.2196/41055

**Published:** 2023-01-20

**Authors:** Neal Yuan, Adam Oesterle, Patrick Botting, Sumeet Chugh, Christine Albert, Joseph Ebinger, David Ouyang

**Affiliations:** 1 Division of Cardiology, Department of Medicine San Francisco Veteran Affairs Medical Center San Francisco, CA United States; 2 Smidt Heart Institute, Department of Cardiology Cedars-Sinai Medical Center Los Angeles, CA United States

**Keywords:** electrocardiogram, QT prolongation, pharmacovigilance, drug toxicity, electronic health records, ECG, EHR, medication monitoring, medication effects, clinical data monitoring, demographic interaction, comorbidity interaction, monitoring, clinical data, accessibility, assessment

## Abstract

**Background:**

Drug-induced prolongation of the corrected QT interval (QTc) increases the risk for Torsades de Pointes (TdP) and sudden cardiac death. Medication effects on the QTc have been studied in controlled settings but may not be well evaluated in real-world settings where medication effects may be modulated by patient demographics and comorbidities as well as the usage of other concomitant medications.

**Objective:**

We demonstrate a new, high-throughput method leveraging electronic health records (EHRs) and the Surescripts pharmacy database to monitor real-world QTc-prolonging medication and potential interacting effects from demographics and comorbidities.

**Methods:**

We included all outpatient electrocardiograms (ECGs) from September 2008 to December 2019 at a large academic medical system, which were in sinus rhythm with a heart rate of 40-100 beats per minute, QRS duration of <120 milliseconds, and QTc of 300-700 milliseconds, determined using the Bazett formula. We used prescription information from the Surescripts pharmacy database and EHR medication lists to classify whether a patient was on a medication during an ECG. Negative control ECGs were obtained from patients not currently on the medication but who had been or would be on that medication within 1 year. We calculated the difference in mean QTc between ECGs of patients who are on and those who are off a medication and made comparisons to known medication TdP risks per the CredibleMeds.org database. Using linear regression analysis, we studied the interaction of patient-level demographics or comorbidities on medication-related QTc prolongation.

**Results:**

We analyzed the effects of 272 medications on 310,335 ECGs from 159,397 individuals. Medications associated with the greatest QTc prolongation were dofetilide (mean QTc difference 21.52, 95% CI 10.58-32.70 milliseconds), mexiletine (mean QTc difference 18.56, 95% CI 7.70-29.27 milliseconds), amiodarone (mean QTc difference 14.96, 95% CI 13.52-16.33 milliseconds), rifaximin (mean QTc difference 14.50, 95% CI 12.12-17.13 milliseconds), and sotalol (mean QTc difference 10.73, 95% CI 7.09-14.37 milliseconds). Several top QT prolonging medications such as rifaximin, lactulose, cinacalcet, and lenalidomide were not previously known but have plausible mechanistic explanations. Significant interactions were observed between demographics or comorbidities and QTc prolongation with many medications, such as coronary disease and amiodarone.

**Conclusions:**

We demonstrate a new, high-throughput technique for monitoring real-world effects of QTc-prolonging medications from readily accessible clinical data. Using this approach, we confirmed known medications for QTc prolongation and identified potential new associations and demographic or comorbidity interactions that could supplement findings in curated databases. Our single-center results would benefit from additional verification in future multisite studies that incorporate larger numbers of patients and ECGs along with more precise medication adherence and comorbidity data.

## Introduction

Prolongation of the corrected QT interval (QTc) increases the risk for malignant ventricular tachyarrhythmias such as Torsades de Pointes (TdP), which can degenerate into ventricular fibrillation and cause sudden cardiac death [1-3]. Medications are the most frequent cause of QTc prolongation and include a number of commonly prescribed drugs such as antiarrhythmics, antipsychotics, antibiotics, and antidepressants [4].

Given the high prevalence of drugs with QTc-prolonging risks, immense effort has been dedicated to maintaining databases of such medications [5]. Currently, most knowledge of the QTc prolonging effects of medications stems from highly controlled individual drug studies as well as regulatory or case reports. However, neither of these approaches offers the ability to systematically monitor QTc prolongation effects in real-world settings, where a medication’s effects may be modulated by patient demographics and comorbidities, as well as with the use of other concomitant medications [6-8].

To address these gaps, we used clinical electrocardiogram (ECG) and electronic health record (EHR) data from a large academic health care system to screen medications for their real-world effects on the QTc. Using this high-throughput approach comparing the same patients before and after initiation of drug therapy, we clarify the associations between medications and QTc prolongation, as well as potential interacting effects from demographics and comorbidities.

## Methods

### Overview

We analyzed QTc durations from all ECGs performed from September 30, 2008, to December 31, 2019, at Cedars-Sinai Medical Center, a large academic medical system in Los Angeles, California, which provides quaternary care and completes more than 800,000 outpatient visits a year. We excluded inpatient ECGs because of high uncertainty and variations in medication history while patients are hospitalized. Given the known limitations of QTc quantification at extremes of interval lengths and heart rate, we included only ECGs in sinus rhythm with a heart rate of 40-100 beats per minute (bpm), QRS duration of <120 milliseconds, and QTc duration between 300 and 700 milliseconds [9,10]. QTc durations were automatically calculated using the Bazett formula [11]. We additionally calculated QTc durations using the Fridericia (QTc = QT / [RR^1/3^]), Hodges (QTc = 0.00175 [60 / RR – 60]), Framingham (QTc = QT + 0.154 [1 – RR]), and Rautaharju (QTc = QT + 0.0185 [RR – 1] + 0.006 for men) formulae [10]. We used medication data from the Surescripts pharmacy database. Surescripts is an electronic prescription clearing house, which routes prescriptions between EHRs and pharmacies, with an estimated coverage of >95% of US pharmacies. We also used medication lists from all outpatient visits and hospitalizations captured by the Epic EHR system during the same time period. We studied the 300 most frequently prescribed medications in the Surescripts database plus all outpatient cardiovascular medications. We further limited our list to only those medications that are systemically active and are not taken on an as-needed basis.

**Figure 1:**
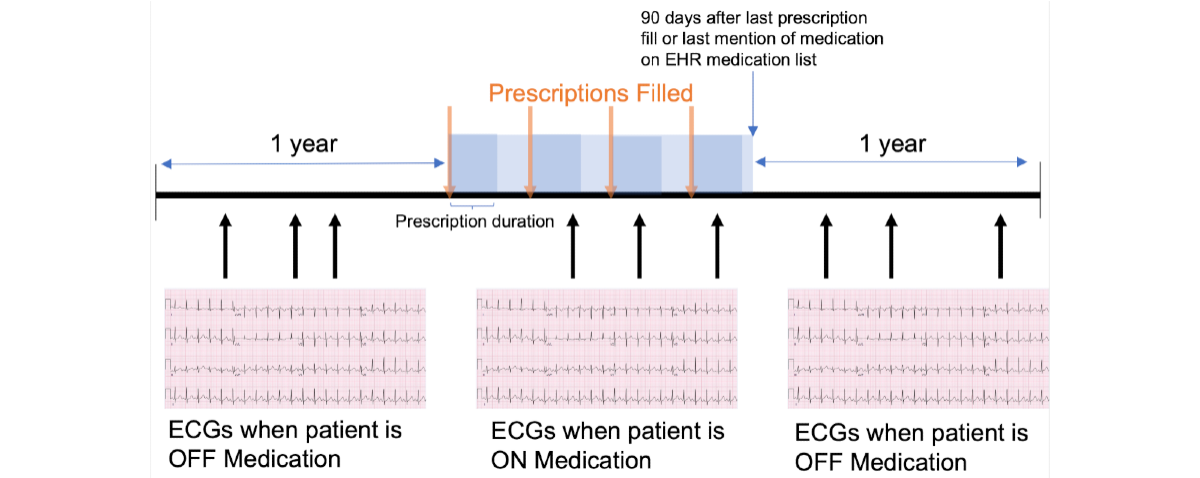
Method for determining electrocardiograms (ECGs) of patients who are on and off a particular medication. EHR: electronic health record.

A patient was considered to be taking a particular medication if the date of the ECG obtained was within a filled medication prescription duration (Figure 1). A patient was considered to not be taking a medication if the ECG date was before the earliest prescription date of that medication or more than 90 days after the last prescription date of the medication. We required that patients “not on a medication” be restricted to only those who had been or would be prescribed the medication within 1 year. This condition was implemented to ensure that ECGs obtained from patients on a medication and those who are off a medication were derived from the same population to reduce confounding by indication. A single ECG could be used for multiple medications given that patients were often taking multiple medications simultaneously at the time of an ECG.

We assessed the difference in the mean QTc duration between ECGs when patients were likely to be taking the medication and those when patients were likely to not be taking the medication. We calculated 95% CIs for QTc differences by bootstrapping with 1000 replications. We visualized medications in accordance with their known risk of TdP per the publicly available database at CredibleMeds.org, which was founded in 2000 with the support of the Agency for Healthcare Research and Quality [5].

We additionally studied the interaction of patient-level demographics and clinical comorbidities with the QTc-prolonging effects of the top QTc-prolonging medications identified by at least 3 of the 5 QTc formulae. Demographics (age, sex, and race and ethnicity) and comorbidities (hypertension, coronary artery disease [CAD], heart failure, diabetes, chronic kidney disease, liver disease, and chronic obstructive pulmonary disease [COPD]) were determined from the EHR. Comorbidities were derived from International Classification of Diseases, Tenth Revision, codes associated with patient visits and problems lists per previously published methods [12,13]. We first performed simple univariate linear regressions modeling the associations of each demographic or comorbidity with QTc. Next, for each medication, we performed linear regressions across each demographic or comorbidity of the following form:

QTc = a + b × medication + c × (demographic or comorbidity) + d × medication × (demographic or comorbidity)

Significant interaction coefficients were displayed using a heat map. All hypothesis testing was 2-sided, and results were evaluated with a significance level of α=.05 after Bonferroni correction for multiple comparisons for each medication.

Data analysis and visualization was performed with R statistical software (version 3.4.1; R Project for Statistical Computing).

### Ethics Approval

This study was approved by the institutional review board of Cedars-Sinai Medical Center (STUDY00001506).

## Results

A total of 310,335 ECGs from 159,397 individuals taking a total of 272 medications met our inclusion criteria (Table 1). The average age of patients at the time of ECG was 59.81 (SD 18.67) years with 44.6% (N=135,364) of ECGs obtained from non-White patients. Patients represented a relatively healthy outpatient cohort, although cardiovascular comorbidities were common, with 23.1% (n=71,715) of participants having hypertension, 10.9% (n=33,900) of them having CAD, 10.3% (n=32,005) of them having diabetes, 7.6% (n=23,570) of them having heart failure, and 8.8% (27,427) chronic kidney disease at the time of ECG. The mean QT was 400.54 (SD 36.05) milliseconds and heart rate was 73.20 bpm (SD 12.56) milliseconds with a QTc of 437.44 (SD 29.41) milliseconds by the Bazett formula.

**Figure 2:**
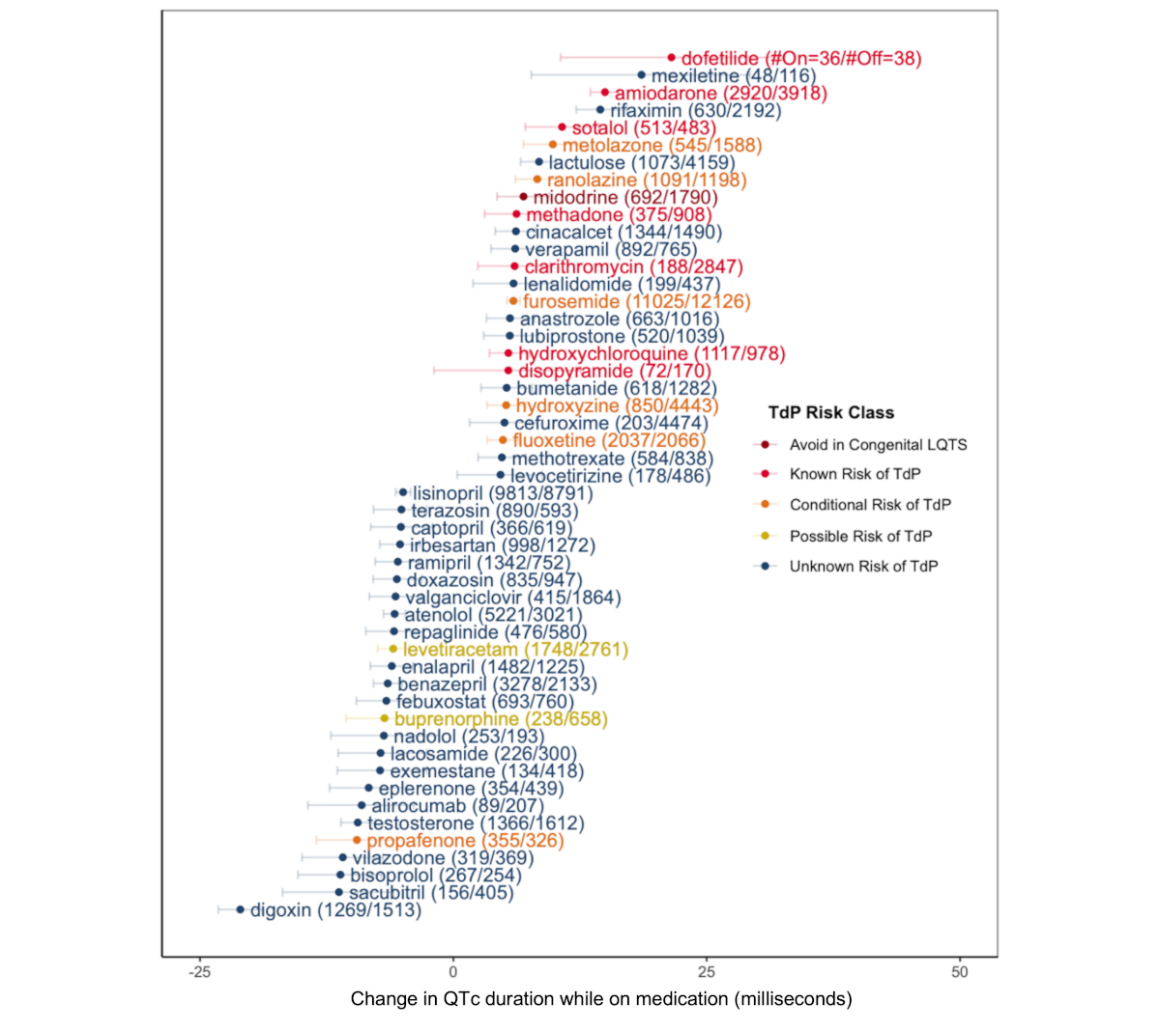
Medications with largest QTc-lengthening and -shortening effects. Mean change in QTc duration while on medication versus while off medication. Intervals represent 95% CIs. TdP risk class is in accordance with classifications by CredibleMeds.org. LQTS: long QT syndrome; TdP: Torsades de Pointes; #On: number of electrocardiograms of patients on medication; #Off: number of electrocardiograms of patients off medication; QTc: corrected QT interval.

We identified medications associated with the greatest change in mean QTc (Figure 2). Overall results across all studied medications are provided in Multimedia Appendix 1. Medications associated with the greatest QTc prolongation when comparing patients on versus those off the medication at the time of ECG included dofetilide (mean QTc difference 21.52 , 95% CI 10.58-32.70 milliseconds), mexiletine (mean QTc difference 18.56, 95% CI 7.70-29.27 milliseconds), amiodarone (mean QTc difference 14.96, 95% CI 13.52-16.33 milliseconds), rifaximin (mean QTc difference 14.50, 95% CI 12.12-17.13 milliseconds), and sotalol (mean QTc difference 10.73, 95% CI 7.09-14.37 milliseconds). Medications associated with the greatest QTc shortening included digoxin (mean QTc difference –21.02, 95% CI –23.20 to –18.85 milliseconds), sacubitril or valsartan (mean QTc difference –11.29, 95% CI –16.86 to –5.90 milliseconds), bisoprolol (mean QTc difference –11.15, 95% CI –15.34 to –7.11 milliseconds), vilazodone (mean QTc difference –10.91, 95% CI –14.94 to –6.92 milliseconds), and propafenone (mean QTc difference –9.52, 95% CI –13.53 to –5.55 milliseconds). Notably, certain identified medications, such as rifaximin, lactulose, cinacalcet, lenalidomide, mercaptopurine, and ritonavir, were not previously known to be associated with QTc prolongation. Our real-world approach was consistent with known classifications, as the average QTc prolongation by TdP class, according to CredibleMeds.org, decreased as expected when comparing across meds with known TdP risk (mean 3.68, SD 5.96 milliseconds) to avoid congenital long QT syndrome (mean 1.71. SD 3.05 milliseconds), conditional risk (mean 1.60, SD 3.75 milliseconds), possible risk (mean 0.65, SD 2.99 milliseconds), and unclassified risk (mean –0.65, SD 4.05 milliseconds).

**Table 1:** Baseline patient and electrocardiogram (N=310,335) characteristics.

Characteristics			Values
Age (years), mean (SD)			59.81 (18.67)
**Sex, n (%)**			
	Female	160,757 (53.5)	
	Male	149,578 (46.5)	
**Race, n (%)**			
	American Indian	540 (0.2)	
	Asian	17,868 (5.8)	
	Black	56,825 (18.3)	
	Hispanic	33,146 (10.7)	
	Non-Hispanic White	174,971 (56.4)	
	Pacific Islander	611 (0.2)	
	Other	26,374 (8.5)	
**Comorbidities, n (%)**			
	Hypertension	71,715 (23.1)	
	Coronary artery disease	33,900 (10.9)	
	Heart failure	23,570 (7.6)	
	Diabetes mellitus	32,005 (10.3)	
	Chronic kidney disease	27,427 (8.8)	
	Liver disease	4803 (1.5)	
	Chronic obstructive pulmonary disease	8349 (2.7)	
QRS duration (milliseconds), mean (SD)			88.14 (11.16)
QT interval (milliseconds), mean (SD)			400.54 (36.05)
Heart rate (beats per minute), mean (SD)			73.20 (12.56)
QTc^a^ Bazett (milliseconds), mean (SD)			437.44 (29.41)
QTc Fridericia (milliseconds), mean (SD)			424.97 (26.78)
QTc Framingham (milliseconds), mean (SD)			400.56 (36.03)
QTc Hodges (milliseconds), mean (SD)			400.56 (36.03)
QTc Rautaharju (milliseconds), mean (SD)			428.13 (26.92)

^a^QTc: corrected QT interval.

**Figure 3:**
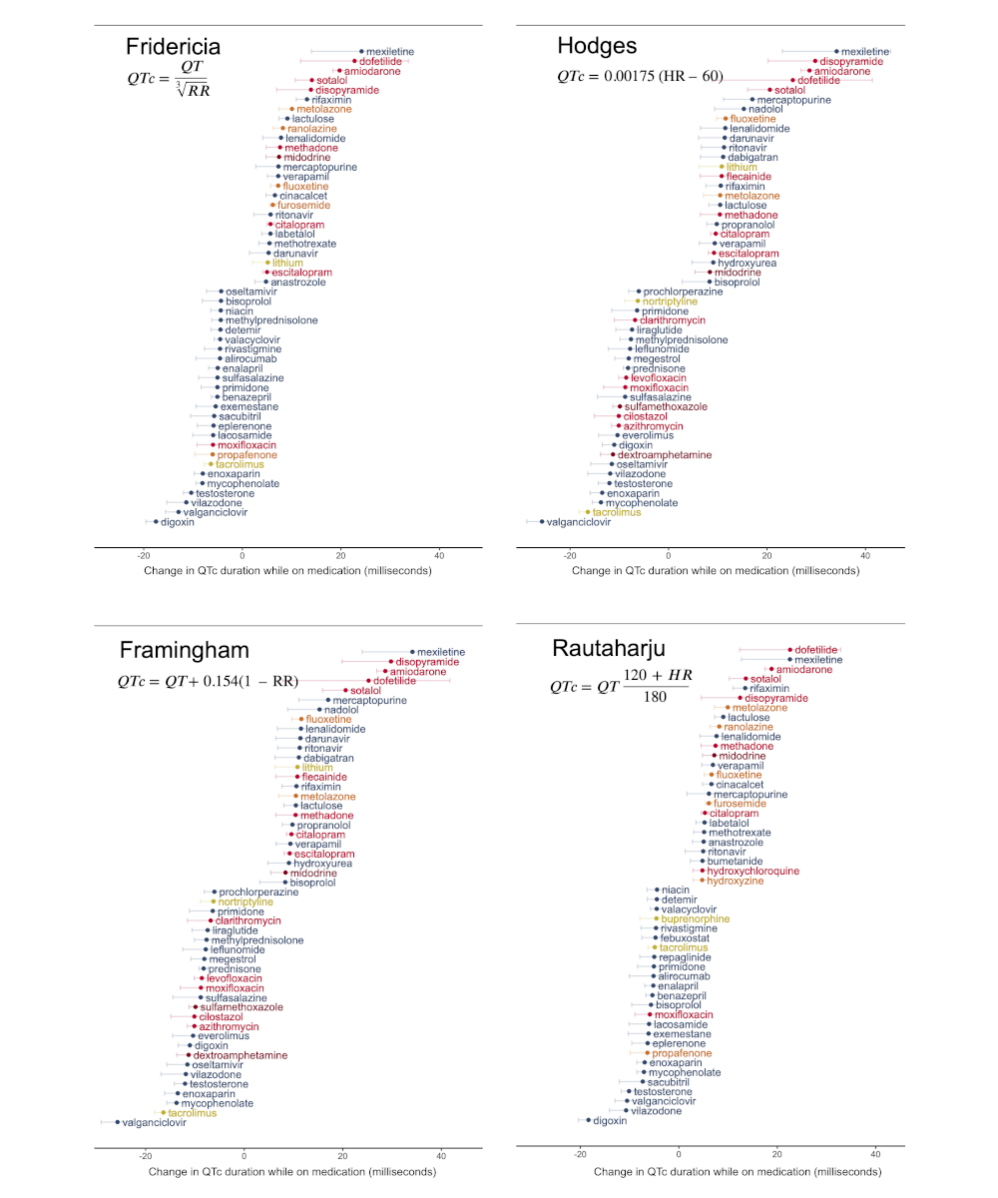
Comparison of medications with largest QT interval (QTc) effects across different QTc formulae.

There was also consistency in medications assessed to have the most QTc prolongation across different QTc assessment methods (Figure 3). However, in contrast to the Bazett, Fridericia, and Rautaharju formulae, the Framingham and Hodges formulae showed no substantial QTc prolongation for several medications known to have a TdP risk.

**Figure 4:**
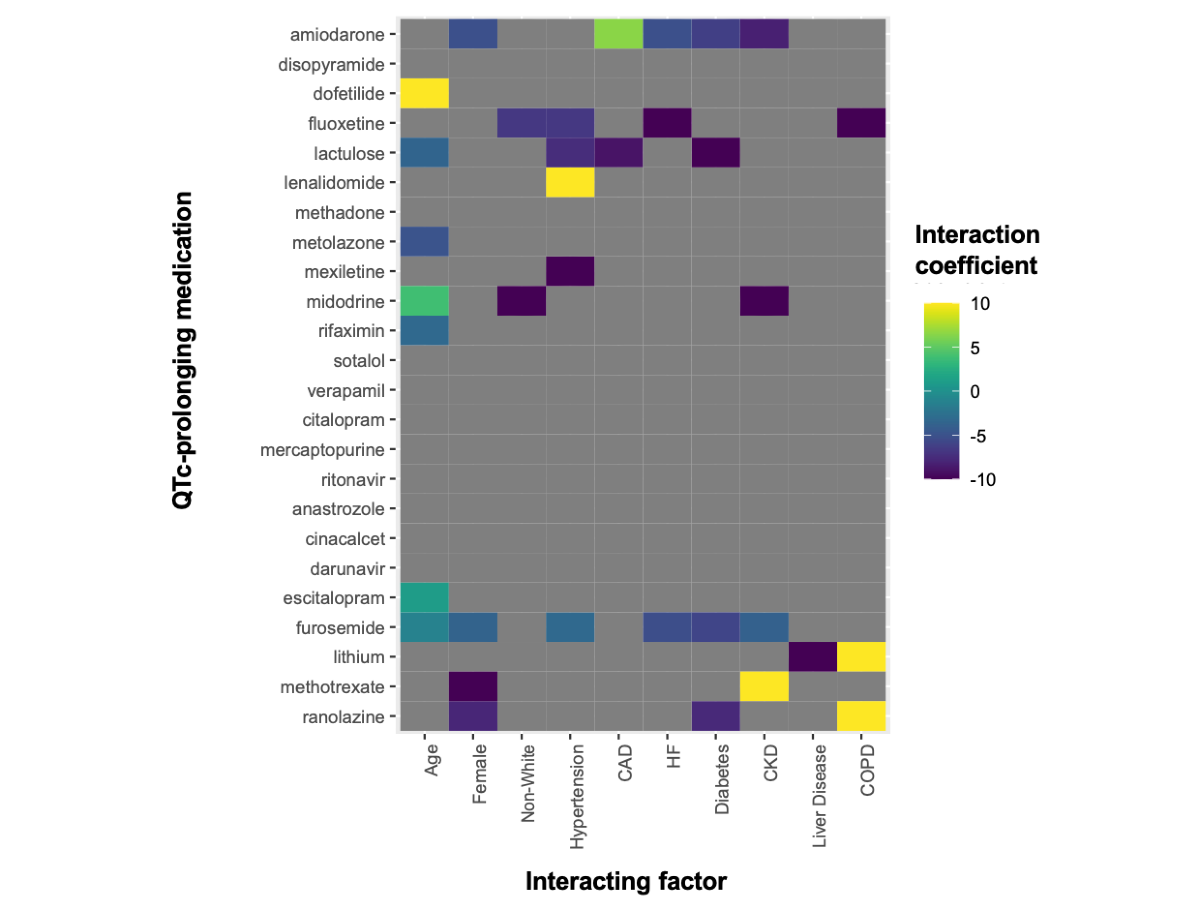
Interaction of demographics and comorbidities on medication with QTc-prolonging effects. For each medication, we performed linear regressions across each demographic or comorbidity of the form: QTc change = a + b × medication + c × demographic/comorbidity + d × medication × demographic/comorbidity. Significant interaction coefficients are displayed using a heat map. All hypothesis testing was 2-sided, and results were evaluated with a significance level of α=.05 after Bonferroni correction for multiple comparisons. CAD: coronary artery disease; CKD: chronic kidney disease; COPD: chronic obstructive pulmonary disease; HF: heart failure; QTc: corrected QT interval.

**Figure 5:**
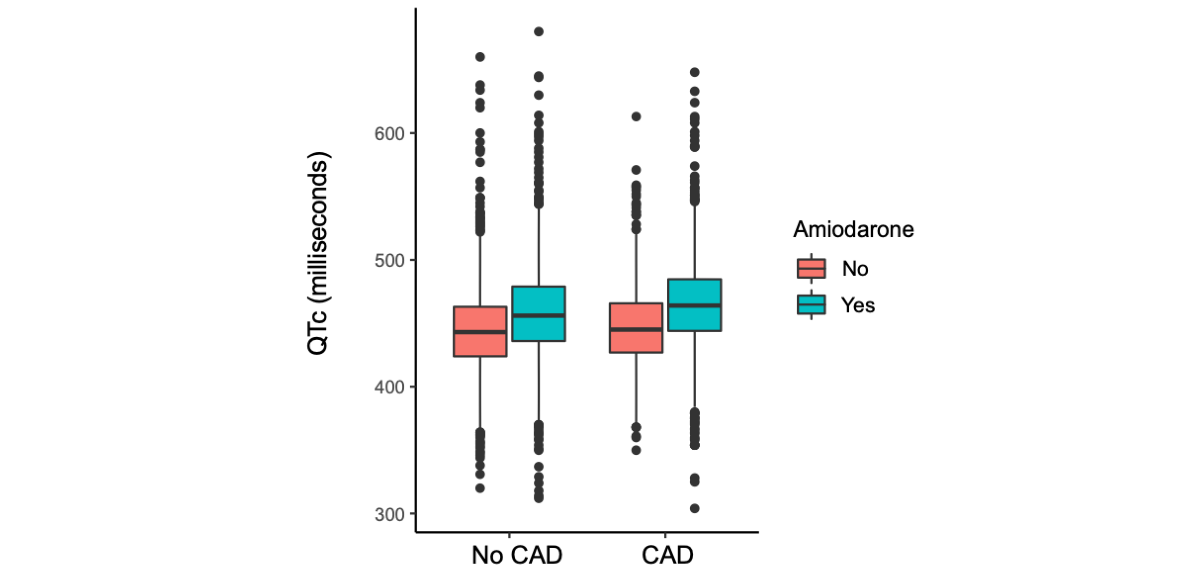
Interaction between amiodarone and CAD on QTc duration. CAD: coronary artery disease; QTc: corrected QT interval.

The QTc was lengthened by all major demographics and comorbidities including age (mean QTc difference 2.5, SD 0.03 milliseconds per 10 years), female sex (mean QTc difference 7.20, SD 0.11 milliseconds), non-White race (mean QTc difference 1.08, SD 0.11 milliseconds), hypertension (mean QTc difference 6.93, SD 0.13 milliseconds), CAD (mean QTc difference 4.70, SD 0.17 milliseconds), heart failure (mean QTc difference 17.1, SD 0.20 milliseconds), diabetes (mean QTc difference 8.57, SD 0.17 milliseconds), chronic kidney disease (mean QTc difference 14.7, SD 0.18 milliseconds), liver disease (mean QTc difference 16.4, SD 0.43 milliseconds), and COPD (mean QTc difference 6.89, SD 0.33 milliseconds). The QTc prolongation effects of several individual medications were significantly modified by demographics and comorbidities (Figure 4). The top 6 positive interactions between demographics or comorbidities and QTc-prolonging medication were between age and dofetilide, hypertension and lenalidomide, chronic kidney disease and methotrexate, COPD and ranolazine, COPD and lithium, and CAD and amiodarone. As one example of these interactions, we show the distribution of QTc duration for patients with and those without CAD before and after taking amiodarone (Figure 5).

Given that QTc can be associated with heart rate, we performed a limited analysis of medications with the most QTc prolongation showing minimal changes in heart rate for most medications when comparing ECGs of patients who are on versus those who are off medication (Multimedia Appendix 2).

## Discussion

### Principal Findings

We demonstrate a new, high-throughput technique for identifying and monitoring real-world medication-related QTc prolongation effects using readily accessible clinical data. Using this approach, we confirmed previously known medications associated with QTc prolongation as well as identified potential new medications not identified in curated databases. While considerable effort has been made to maintain well-researched lists of potentially dangerous medications using data from highly controlled studies, little has been published about the real-world effects of medications on QTc duration. It is well known that demographics, comorbidities, and drug-drug interactions may have profound effects on QTc but may not be represented in conventional drug study data [6-8]. We envision that applying our described technique across multiple centers could be used for (1) identifying high-TdP-risk medications that may have little prior data but require further study, (2) confirming or disputing case reports of medications associated with TdP, and (3) understanding how individual drug QTc prolongation effects may be modulated by demographics, comorbidities, and other medications.

### Comparison to Prior Work

Our analysis was able to distinguish drugs with known QTc-prolonging effects, including antiarrhythmics (dofetilide, amiodarone, sotalol, and disopyramide), antibiotics (clarithromycin), and antidepressants (fluoxetine, citalopram, and escitalopram). Interestingly, we also identified medications such as rifaximin, lactulose, cinacalcet, lenalidomide, mercaptopurine, and ritonavir, for which there are few data about QTc prolonging effects, but which may deserve additional study. Cinacalcet, for example, has been shown, in very small-scale studies, to be associated with QTc prolongation beyond what might be expected from chronic kidney disease, possibly through its effects on serum calcium levels [14,15]. Lenalidomide, a drug used to treat hematologic malignancies often in older patients with comorbidities, has known cardiovascular toxicities but has only been studied on the basis of its effects on ECGs in 60 healthy, young male volunteers [16,17]; it has also been understudied in Black individuals [18]. Other medications such as mercaptopurine were introduced to drug formularies long before drug trials with QTc prolongation safety analyses were standard of practice. It is also possible that the QTc prolongation observed with some medications such as rifaximin and lactulose may reflect underlying comorbidities such as liver disease, which may prolong the QTc [19,20]. Other medications, such as ritonavir and verapamil, are cytochrome P450 3A4 isozyme inhibitors and may have QTc effects via prominent drug-drug interactions. We did note that mexiletine, a sodium channel blocker known to shorten the QTc and used to treat congenital long QT syndrome type 3, was associated with QTc prolongation. This may be due to mexiletine being prescribed either in combination with QTc-prolonging agents such as amiodarone or as treatment for patients with prolonged QTc [21]. In our cohort, 27 of 48 (57%) patients taking mexiletine were also taking amiodarone, and a simple linear regression revealed that the QTc-prolonging effect of mexiletine was not significant after accounting for amiodarone.

We found that our technique was relatively uninfluenced by the QTc assessment method in identifying medications at the highest risk for QTc prolongation. While prior studies suggested that some methods may perform better at certain heart rate ranges or may better predict even mortality, we found that the effects of QTc-prolonging medications were still observable regardless of the QTc method used [9,10]. This may have been partially due to our deliberate choice to limit our cohort of ECGs to those with a heart rate between 40 and 100 bpm. We did find, however, that the Hodges and Framingham methods, which are known to underestimate QTc prolongation relative to the Bazett formula, seemed to underdetect QTc prolongation with several medications that have known QT-prolonging effects [10].

Demographics and comorbidities all increased the average QTc duration. Consistent with risk factors that are included in the frequently used Tisdale Risk Score for QTc prolongation, we found that age, female sex, heart failure, and CAD were associated with particularly significant increases in QTc [8]. Liver disease and chronic kidney disease were also notably associated with prominent QTc increases. Demographics and comorbidities, not surprisingly, therefore had significant modifying influences on the QTc prolonging effects of many individual medications. While on average, QTc increases with age, age was additionally associated with increased QTc prolongation of dofetilide and decreased QTc prolongation from metolazone, lactulose, or rifaximin [22]. Women, on average, have longer QTcs and a higher risk of experiencing TdP while on QTc-prolonging medications [23,24]. Tisdale et al [8] found that female sex had an odds ratio of 1.5 for QTc prolongation (defined as a QTc of >500 milliseconds or an increase of >60 milliseconds). Interestingly, we observed that in women, there may actually be less change in QTc associated with several of our identified medications. It could be that even if some medications do not change the QTc by as much in female patients, women on average start with a longer QTc and therefore still end up more frequently with a QTc of >500 milliseconds. Race was also a significant interacting factor, which adds emphasis to potential gaps in our current understanding of the QTc-prolonging effects of medications in non-White populations due to lack of racial diversity in drug studies [25,26]. With regard to the effects of comorbidities, all of the major chronic conditions studied were associated with interacting effects with certain medications. This may be due to altered medication metabolism or electrolyte disturbances in cases of kidney or liver disease or due to other interacting effects such as autonomic modulation that may affect QTc duration in patients with COPD [27]. The QTc-prolonging effects of amiodarone were different in patients with coronary disease and those with heart failure, which suggest the influence of cardiac structural change on drug-induced QTc prolongation.

### Limitations and Future Directions

Several limitations of this study warrant consideration. Although QTc prolongation correlates with TdP risk, prolongation in itself is not fully predictive of TdP. As such, identifying medication that prolong QTc is likely a first step in truly understanding a medication’s effects on TdP risk. While we believe that our method for identifying whether a patient was on or off a medication was quite rigorous, using both Surescripts pharmacy and EHR data, medication adherence remains challenging to assess, and patients could still receive prescriptions from external sources. Both of these cases (nonadherence and external prescription) would bias our results to the null. We pooled ECGs from patients both before as well as after they were on medication to create our off-medication cohorts. This increased the number of ECGs that could be used and did not significantly change whether a medication prolonged the QTc when compared to using only ECGs from patients before starting a medication (Multimedia Appendix 3). However, future studies could consider limiting the off-medication cohort to only ECGs from patients before starting a medication. As this was a relatively healthy population, some of the interaction effects between less common comorbidities (eg, liver disease) and medications (eg, dofetilide, disopyramide, and mexiletine) may be less reliable and should be confirmed in cohorts with larger numbers. We additionally acknowledge that there can be inaccuracies in identifying comorbidities and demographics when relying on EHR data and International Classification of Diseases, Tenth Revision, coding. Future studies using more rigorously adjudicated registries could add precision. Lastly, there remains many possible co-occurring confounding reasons for a medication to be associated with QTc prolongation: patient demographics, comorbidities, electrolyte abnormalities, and drug-drug interactions. However, even if a certain medication tends to be used among patients with other QTc-prolonging risk factors, we still hold that such information is useful as a real-world reflection of QTc prolongation risk. To control for some of these confounders, we ensured that for each medication, ECGs were compared only among patients who were prescribed that medication at some point in time, thereby ensuring that ECGs of patients on and those of patients off the medication were drawn from the same patient population. Nevertheless, the associations uncovered by our methodology are meant to be exploratory and require dedicated prospective studies for confirmation.

### Conclusions

In this study, we demonstrate a high-throughput method using accessible clinical data to identify and monitor the real-world QTc prolongation associations of all commonly prescribed medications. Such a technique might be easily deployable across multiple medical centers for identifying and confirming suspected medications with QTc-prolonging risks and the demographic and comorbidity factors that may enhance or mitigate such risks.

## Appendix

Multimedia Appendix 1 QTc changes (ms) across all medications studied.

Multimedia Appendix 2 Change in heart rate when comparing ECGs on versus off of medication.

Multimedia Appendix 3 Changes in QTc when on a medication compared to when off a medication before or after being on the medication.

## Abbreviations

bpm: beats per minute
CAD: coronary artery disease
COPD: chronic obstructive pulmonary disease
ECG: electrocardiogram
EHR: electronic health records
QTc: corrected QT interval
TdP: Torsades de Pointes

## Data Availability

Given the potential identifying nature of the intersection of a patient’s ECG, demographics, comorbidities, and medications, the entire data set will not be made publicly available. However, a limited subset of deidentified data may be made available upon reasonable request to the corresponding author.
