# Supplementary material for: High-Throughput Assessment of Real-World Medication Effects on QT Interval Prolongation: Observational Study
Source: JMIR Cardio. 2023 Jan 20;7:e41055. doi: 10.2196/41055 (PMC9898836; doi:10.2196/41055)
Supplement: Multimedia Appendix 1 [file cardio_v7i1e41055_app1.docx]

*Supplemental Table 1. QTc changes (ms) across all medications studied*

| **Medication** | **QTcDiff Bazett** | **QTcDiff Fridericia** | **QTcDiff  Framingham** | **QTcDiff Hodges** | **QTcDiff Rautaharju** |
| --- | --- | --- | --- | --- | --- |
| dofetilide | 21.5 | 22.8 | 25.2 | 25.2 | 22.6 |
| mexiletine | 18.6 | 24.2 | 34.1 | 34.1 | 22.6 |
| amiodarone | 15.0 | 19.7 | 28.6 | 28.6 | 18.8 |
| rifaximin | 14.5 | 13.1 | 10.6 | 10.6 | 13.5 |
| sotalol | 10.7 | 14.1 | 20.6 | 20.6 | 13.6 |
| metolazone | 9.8 | 10.1 | 10.5 | 10.5 | 9.9 |
| lactulose | 8.5 | 9.1 | 10.5 | 10.5 | 9.0 |
| ranolazine | 8.3 | 8.2 | 8.1 | 8.1 | 8.2 |
| midodrine | 6.9 | 7.4 | 8.4 | 8.4 | 7.2 |
| methadone | 6.2 | 7.7 | 10.4 | 10.4 | 7.5 |
| cinacalcet | 6.2 | 6.6 | 7.4 | 7.4 | 6.6 |
| verapamil | 6.1 | 7.3 | 9.4 | 9.4 | 6.9 |
| clarithromycin | 6.0 | 1.5 | -6.8 | -6.8 | 2.6 |
| lenalidomide | 5.9 | 7.8 | 11.5 | 11.5 | 7.7 |
| furosemide | 5.9 | 6.2 | 6.7 | 6.7 | 6.1 |
| anastrozole | 5.6 | 4.8 | 3.4 | 3.4 | 5.0 |
| lubiprostone | 5.6 | 3.4 | -0.5 | -0.5 | 4.0 |
| hydroxychloroquine | 5.4 | 4.7 | 3.3 | 3.3 | 4.8 |
| disopyramide | 5.4 | 13.9 | 29.8 | 29.8 | 12.4 |
| bumetanide | 5.3 | 4.6 | 3.2 | 3.2 | 4.8 |
| hydroxyzine | 5.2 | 4.6 | 3.5 | 3.5 | 4.7 |
| cefuroxime | 5.0 | 3.3 | 0.3 | 0.3 | 3.7 |
| fluoxetine | 4.9 | 7.3 | 11.6 | 11.6 | 6.6 |
| methotrexate | 4.8 | 5.5 | 6.7 | 6.7 | 5.1 |
| levocetirizine | 4.7 | 4.2 | 3.3 | 3.3 | 4.2 |
| labetalol | 4.5 | 5.7 | 7.8 | 7.8 | 5.2 |
| modafinil | 4.5 | 2.6 | -0.9 | -0.9 | 3.1 |
| torsemide | 4.2 | 4.2 | 4.3 | 4.3 | 4.1 |
| icosapent | 4.0 | 3.9 | 3.9 | 3.9 | 4.1 |
| imipramine | 4.0 | 1.5 | -3.2 | -3.2 | 2.0 |
| dulaglutide | 4.0 | 2.3 | -0.8 | -0.8 | 2.6 |
| lisdexamfetamine | 3.9 | 1.8 | -2.2 | -2.2 | 2.1 |
| duloxetine | 3.9 | 2.3 | -0.7 | -0.7 | 2.5 |
| armodafinil | 3.8 | 1.9 | -1.6 | -1.6 | 2.2 |
| potassium | 3.8 | 4.3 | 5.3 | 5.3 | 4.2 |
| citalopram | 3.6 | 5.7 | 9.6 | 9.6 | 5.3 |
| glyburide | 3.6 | 3.6 | 3.5 | 3.5 | 3.5 |
| loratadine | 3.3 | 2.8 | 1.6 | 1.6 | 2.8 |
| cholecalciferol | 3.3 | 2.9 | 2.2 | 2.2 | 3.1 |
| cetirizine | 3.2 | 3.0 | 2.6 | 2.6 | 2.9 |
| linaclotide | 3.1 | 3.2 | 3.4 | 3.4 | 3.2 |
| memantine | 3.1 | 4.0 | 5.9 | 5.9 | 3.9 |
| oxycodone | 3.0 | 0.5 | -4.2 | -4.2 | 1.1 |
| nortriptyline | 3.0 | -0.3 | -6.2 | -6.2 | 0.6 |
| ciprofloxacin | 2.9 | 1.8 | -0.3 | -0.3 | 2.0 |
| sirolimus | 2.9 | 2.0 | 0.3 | 0.3 | 2.3 |
| meclizine | 2.9 | 4.2 | 6.6 | 6.6 | 4.0 |
| acetaminophen | 2.9 | 0.9 | -3.0 | -3.0 | 1.3 |
| escitalopram | 2.8 | 5.0 | 9.2 | 9.2 | 4.6 |
| ritonavir | 2.7 | 5.7 | 11.3 | 11.3 | 4.9 |
| venlafaxine | 2.6 | 1.6 | -0.5 | -0.5 | 1.9 |
| aspirin | 2.6 | 1.7 | -0.1 | -0.1 | 1.9 |
| hydrocodone | 2.6 | 0.6 | -3.3 | -3.3 | 1.0 |
| olanzapine | 2.6 | 0.7 | -2.8 | -2.8 | 1.2 |
| alfuzosin | 2.6 | 1.4 | -0.7 | -0.7 | 1.9 |
| ursodiol | 2.5 | 3.4 | 5.0 | 5.0 | 3.1 |
| tolterodine | 2.5 | 3.2 | 4.5 | 4.5 | 3.2 |
| metoclopramide | 2.5 | 0.7 | -2.7 | -2.7 | 1.2 |
| topiramate | 2.4 | 2.2 | 1.9 | 1.9 | 2.3 |
| letrozole | 2.3 | 1.6 | 0.1 | 0.1 | 1.7 |
| azathioprine | 2.3 | 3.2 | 4.7 | 4.7 | 3.0 |
| paroxetine | 2.3 | 3.9 | 6.9 | 6.9 | 3.7 |
| esomeprazole | 2.3 | 2.1 | 1.8 | 1.8 | 2.1 |
| magnesium | 2.3 | 2.2 | 2.1 | 2.1 | 2.2 |
| ondansetron | 2.2 | 0.3 | -3.2 | -3.2 | 0.8 |
| clonazepam | 2.2 | 3.4 | 5.5 | 5.5 | 3.1 |
| leflunomide | 2.2 | -1.3 | -7.8 | -7.8 | -0.6 |
| amitriptyline | 2.2 | -0.5 | -5.3 | -5.3 | 0.2 |
| hydralazine | 2.2 | 3.7 | 6.4 | 6.4 | 3.3 |
| ofloxacin | 2.2 | -0.1 | -4.2 | -4.2 | 0.4 |
| mercaptopurine | 2.1 | 7.3 | 17.0 | 17.0 | 6.1 |
| lithium | 2.1 | 5.2 | 10.8 | 10.8 | 4.4 |
| oxybutynin | 2.0 | 1.9 | 1.6 | 1.6 | 1.9 |
| pantoprazole | 2.0 | 1.3 | 0.0 | 0.0 | 1.5 |
| zaleplon | 2.0 | 0.9 | -1.0 | -1.0 | 1.2 |
| ropinirole | 2.0 | 1.4 | 0.4 | 0.4 | 1.6 |
| darunavir | 1.9 | 5.3 | 11.4 | 11.4 | 4.2 |
| raloxifene | 1.9 | 1.6 | 0.8 | 0.8 | 1.6 |
| emtricitabine | 1.9 | 2.7 | 4.2 | 4.2 | 2.6 |
| risedronate | 1.9 | 2.8 | 4.7 | 4.7 | 2.7 |
| tamoxifen | 1.9 | 2.1 | 2.4 | 2.4 | 2.0 |
| isosorbide | 1.8 | 3.5 | 6.5 | 6.5 | 3.2 |
| gabapentin | 1.8 | 1.7 | 1.5 | 1.5 | 1.8 |
| celecoxib | 1.8 | 2.3 | 3.1 | 3.1 | 2.1 |
| pregabalin | 1.7 | 1.8 | 1.9 | 1.9 | 1.7 |
| prochlorperazine | 1.7 | -1.0 | -6.1 | -6.1 | -0.4 |
| cyclobenzaprine | 1.6 | 0.0 | -2.9 | -2.9 | 0.4 |
| solifenacin | 1.6 | 1.3 | 0.5 | 0.5 | 1.3 |
| degludec | 1.6 | 2.2 | 3.3 | 3.3 | 2.1 |
| nitrofurantoin | 1.5 | 1.1 | 0.7 | 0.7 | 1.3 |
| vortioxetine | 1.4 | 1.4 | 1.4 | 1.4 | 1.4 |
| alprazolam | 1.3 | 1.0 | 0.4 | 0.4 | 1.0 |
| diazepam | 1.3 | 0.8 | 0.0 | 0.0 | 1.0 |
| desvenlafaxine | 1.3 | 2.4 | 4.4 | 4.4 | 2.2 |
| liraglutide | 1.3 | -1.8 | -7.4 | -7.4 | -1.2 |
| doxepin | 1.2 | 1.8 | 2.9 | 2.9 | 1.6 |
| quetiapine | 1.2 | 1.1 | 1.0 | 1.0 | 1.2 |
| lorazepam | 1.2 | 0.5 | -0.8 | -0.8 | 0.6 |
| tramadol | 1.2 | 0.8 | 0.1 | 0.1 | 0.9 |
| lurasidone | 1.2 | 0.7 | 0.1 | 0.1 | 1.0 |
| dexlansoprazole | 1.2 | 0.6 | -0.5 | -0.5 | 0.8 |
| amoxicillin | 1.1 | 0.2 | -1.6 | -1.6 | 0.3 |
| oxcarbazepine | 1.1 | 0.5 | -0.7 | -0.7 | 0.5 |
| prednisone | 1.0 | -2.3 | -8.2 | -8.2 | -1.4 |
| divalproex | 1.0 | 1.6 | 2.7 | 2.7 | 1.4 |
| fluconazole | 0.9 | -1.1 | -4.8 | -4.8 | -0.6 |
| linagliptin | 0.9 | -0.1 | -1.7 | -1.7 | 0.2 |
| glargine | 0.9 | 1.6 | 3.1 | 3.1 | 1.4 |
| prasugrel | 0.9 | 3.0 | 7.1 | 7.1 | 2.9 |
| trazodone | 0.8 | 1.1 | 1.6 | 1.6 | 1.0 |
| cephalexin | 0.8 | 0.6 | 0.1 | 0.1 | 0.6 |
| rosuvastatin | 0.8 | 1.8 | 3.5 | 3.5 | 1.5 |
| clonidine | 0.8 | 2.6 | 5.9 | 5.9 | 2.2 |
| quinapril | 0.8 | 0.9 | 1.3 | 1.3 | 0.9 |
| colchicine | 0.7 | -0.1 | -1.4 | -1.4 | 0.1 |
| pitavastatin | 0.7 | 3.2 | 7.8 | 7.8 | 2.7 |
| azilsartan | 0.6 | 1.2 | 2.2 | 2.2 | 1.2 |
| dutasteride | 0.6 | 0.7 | 0.8 | 0.8 | 0.5 |
| sertraline | 0.6 | 2.8 | 6.8 | 6.8 | 2.4 |
| levofloxacin | 0.6 | -2.7 | -8.6 | -8.6 | -1.9 |
| diphenhydramine | 0.6 | -0.6 | -2.7 | -2.7 | -0.2 |
| fluticasone | 0.5 | -0.6 | -2.8 | -2.8 | -0.4 |
| rivaroxaban | 0.5 | 2.8 | 7.2 | 7.2 | 2.5 |
| alendronate | 0.5 | 1.7 | 3.9 | 3.9 | 1.4 |
| dicyclomine | 0.5 | -0.4 | -2.0 | -2.0 | -0.2 |
| donepezil | 0.4 | 2.2 | 5.6 | 5.6 | 1.9 |
| lovastatin | 0.4 | 1.4 | 3.1 | 3.1 | 1.1 |
| megestrol | 0.4 | -2.6 | -8.1 | -8.1 | -1.9 |
| minoxidil | 0.3 | 0.6 | 1.1 | 1.1 | 0.5 |
| phenytoin | 0.3 | 1.6 | 3.9 | 3.9 | 1.2 |
| felodipine | 0.3 | -0.2 | -0.8 | -0.8 | 0.3 |
| pravastatin | 0.3 | -0.7 | -2.4 | -2.4 | -0.3 |
| bupropion | 0.3 | -1.1 | -3.6 | -3.6 | -0.8 |
| ranitidine | 0.2 | 0.5 | 1.1 | 1.1 | 0.5 |
| naproxen | 0.2 | 0.1 | -0.2 | -0.2 | 0.1 |
| flecainide | 0.2 | 3.9 | 10.8 | 10.8 | 3.3 |
| dronedarone | 0.2 | 1.8 | 4.9 | 4.9 | 1.5 |
| azithromycin | 0.1 | -3.5 | -10.1 | -10.1 | -2.6 |
| baclofen | 0.1 | -0.2 | -0.6 | -0.6 | -0.1 |
| everolimus | 0.0 | -3.6 | -10.4 | -10.4 | -2.6 |
| ezetimibe | 0.0 | 1.4 | 4.1 | 4.1 | 1.3 |
| ferrous | 0.0 | 0.2 | 0.5 | 0.5 | 0.1 |
| meloxicam | -0.1 | -0.4 | -1.0 | -1.0 | -0.3 |
| lispro | -0.1 | -0.3 | -0.7 | -0.7 | -0.2 |
| mirtazapine | -0.1 | 0.2 | 0.7 | 0.7 | 0.1 |
| dextroamphetamine | -0.1 | -4.1 | -11.3 | -11.3 | -3.1 |
| metronidazole | -0.1 | -0.2 | -0.4 | -0.4 | -0.2 |
| famotidine | -0.1 | 0.9 | 2.9 | 2.9 | 0.7 |
| canagliflozin | -0.2 | -0.1 | 0.1 | 0.1 | -0.1 |
| methylphenidate | -0.3 | 1.1 | 3.5 | 3.5 | 0.8 |
| clopidogrel | -0.3 | 0.0 | 0.5 | 0.5 | -0.1 |
| cyclosporine | -0.3 | -1.6 | -4.1 | -4.1 | -1.3 |
| oseltamivir | -0.3 | -4.3 | -11.5 | -11.5 | -3.3 |
| eszopiclone | -0.3 | 1.4 | 4.7 | 4.7 | 1.1 |
| glimepiride | -0.4 | -0.9 | -2.0 | -2.0 | -1.0 |
| adalimumab | -0.4 | 1.5 | 5.2 | 5.2 | 1.3 |
| aripiprazole | -0.4 | -0.2 | 0.1 | 0.1 | -0.2 |
| quinidine | -0.5 | 1.0 | 3.5 | 3.5 | 0.5 |
| amlodipine | -0.6 | 0.0 | 1.0 | 1.0 | -0.1 |
| methimazole | -0.6 | 2.0 | 6.9 | 6.9 | 1.4 |
| aspart | -0.6 | -0.5 | -0.2 | -0.2 | -0.4 |
| prednisolone | -0.6 | -1.6 | -3.5 | -3.5 | -1.6 |
| evolocumab | -0.7 | -1.0 | -1.2 | -1.2 | -0.7 |
| mirabegron | -0.7 | -1.8 | -3.9 | -3.9 | -1.5 |
| sulfamethoxazole | -0.7 | -4.0 | -9.9 | -9.9 | -3.1 |
| finasteride | -0.8 | 0.6 | 3.1 | 3.1 | 0.3 |
| cilostazol | -0.8 | -4.1 | -10.1 | -10.1 | -3.1 |
| tamsulosin | -0.8 | 0.1 | 1.8 | 1.8 | 0.0 |
| metoprolol | -0.8 | 1.7 | 6.3 | 6.3 | 1.2 |
| methocarbamol | -0.8 | -0.5 | 0.3 | 0.3 | -0.4 |
| tacrolimus | -0.9 | -6.4 | -16.4 | -16.4 | -4.9 |
| propranolol | -0.9 | 2.8 | 9.8 | 9.8 | 2.1 |
| pioglitazone | -0.9 | -0.7 | -0.1 | -0.1 | -0.6 |
| buspirone | -0.9 | -0.3 | 0.9 | 0.9 | -0.5 |
| rabeprazole | -0.9 | -2.6 | -5.7 | -5.7 | -2.1 |
| warfarin | -1.0 | 0.3 | 2.5 | 2.5 | -0.2 |
| carbamazepine | -1.0 | -1.0 | -0.7 | -0.7 | -0.8 |
| simvastatin | -1.2 | -0.1 | 1.9 | 1.9 | -0.3 |
| erythromycin | -1.2 | -2.6 | -5.3 | -5.3 | -2.4 |
| cyanocobalamin | -1.2 | -0.1 | 1.9 | 1.9 | -0.4 |
| lamotrigine | -1.3 | -0.5 | 1.0 | 1.0 | -0.6 |
| raltegravir | -1.3 | 0.0 | 2.5 | 2.5 | -0.1 |
| nifedipine | -1.3 | -1.6 | -2.2 | -2.2 | -1.6 |
| acyclovir | -1.3 | -1.9 | -3.1 | -3.1 | -1.8 |
| hydrochlorothiazide | -1.3 | -0.4 | 1.4 | 1.4 | -0.6 |
| glipizide | -1.4 | -0.7 | 0.6 | 0.6 | -0.9 |
| ergocalciferol | -1.4 | -0.6 | 0.8 | 0.8 | -0.9 |
| temazepam | -1.4 | -0.8 | 0.3 | 0.3 | -0.9 |
| carbidopa | -1.4 | -1.3 | -1.0 | -1.0 | -1.4 |
| pramipexole | -1.5 | -1.3 | -1.2 | -1.2 | -1.3 |
| ticagrelor | -1.5 | -0.4 | 1.7 | 1.7 | -0.6 |
| chlorthalidone | -1.5 | -0.8 | 0.5 | 0.5 | -0.9 |
| ibandronate | -1.6 | -1.2 | -0.4 | -0.4 | -1.2 |
| etanercept | -1.6 | 0.8 | 5.0 | 5.0 | 0.1 |
| progesterone | -1.7 | 0.0 | 3.1 | 3.1 | -0.3 |
| allopurinol | -1.7 | -0.6 | 1.5 | 1.5 | -0.9 |
| doxycycline | -1.7 | -2.9 | -5.2 | -5.2 | -2.7 |
| estradiol | -1.7 | -1.7 | -1.8 | -1.8 | -1.7 |
| atorvastatin | -1.7 | 0.0 | 3.1 | 3.1 | -0.4 |
| zolpidem | -1.8 | -0.9 | 0.6 | 0.6 | -1.2 |
| nebivolol | -1.8 | 0.9 | 6.1 | 6.1 | 0.4 |
| risperidone | -2.0 | -1.5 | -0.3 | -0.3 | -1.3 |
| denosumab | -2.1 | -1.3 | 0.2 | 0.2 | -1.4 |
| nateglinide | -2.1 | -1.1 | 0.6 | 0.6 | -1.4 |
| ibuprofen | -2.1 | -2.4 | -2.8 | -2.8 | -2.3 |
| morphine | -2.2 | -3.6 | -6.0 | -6.0 | -3.1 |
| clindamycin | -2.2 | -1.9 | -1.3 | -1.3 | -1.9 |
| carvedilol | -2.3 | 0.7 | 6.1 | 6.1 | -0.1 |
| indomethacin | -2.4 | -3.5 | -5.7 | -5.7 | -3.4 |
| dolutegravir | -2.4 | 0.1 | 4.5 | 4.5 | -0.6 |
| levothyroxine | -2.4 | -1.3 | 0.8 | 0.8 | -1.6 |
| diltiazem | -2.5 | -1.4 | 0.5 | 0.5 | -1.8 |
| metformin | -2.5 | -1.9 | -0.8 | -0.8 | -2.1 |
| estrogen | -2.5 | -1.7 | -0.2 | -0.2 | -1.8 |
| cefdinir | -2.6 | -2.7 | -2.9 | -2.9 | -2.5 |
| methylprednisolone | -2.6 | -4.4 | -7.6 | -7.6 | -4.1 |
| empagliflozin | -2.8 | -1.3 | 1.3 | 1.3 | -1.6 |
| dapagliflozin | -2.8 | -0.3 | 4.4 | 4.4 | -0.8 |
| spironolactone | -2.8 | -1.6 | 0.8 | 0.8 | -1.9 |
| mesalamine | -2.9 | -0.4 | 4.4 | 4.4 | -0.9 |
| losartan | -2.9 | -1.7 | 0.7 | 0.7 | -2.0 |
| hydroxyurea | -2.9 | 1.2 | 9.1 | 9.1 | 0.5 |
| sulfasalazine | -3.0 | -5.0 | -8.8 | -8.8 | -4.4 |
| dexamethasone | -3.0 | -3.8 | -5.3 | -5.3 | -3.6 |
| rasagiline | -3.0 | -2.9 | -3.3 | -3.3 | -3.6 |
| liothyronine | -3.1 | -2.1 | -0.4 | -0.4 | -2.5 |
| sitagliptin | -3.4 | -2.8 | -1.8 | -1.8 | -3.0 |
| methenamine | -3.5 | -1.4 | 2.5 | 2.5 | -1.9 |
| acetazolamide | -3.6 | -3.4 | -3.1 | -3.1 | -3.5 |
| indapamide | -3.6 | -3.5 | -3.3 | -3.3 | -3.5 |
| olmesartan | -3.7 | -3.2 | -2.2 | -2.2 | -3.3 |
| apixaban | -3.9 | 0.2 | 7.9 | 7.9 | -0.4 |
| silodosin | -3.9 | -3.3 | -2.5 | -2.5 | -3.8 |
| detemir | -4.2 | -4.4 | -5.1 | -5.1 | -4.5 |
| calcitriol | -4.2 | -3.8 | -3.0 | -3.0 | -3.8 |
| fenofibrate | -4.2 | -2.2 | 1.4 | 1.4 | -2.7 |
| valacyclovir | -4.3 | -4.5 | -4.8 | -4.8 | -4.5 |
| rivastigmine | -4.4 | -4.5 | -4.9 | -4.9 | -4.6 |
| primidone | -4.4 | -5.1 | -6.4 | -6.4 | -5.1 |
| niacin | -4.5 | -4.4 | -4.1 | -4.1 | -4.4 |
| moxifloxacin | -4.5 | -6.0 | -8.8 | -8.8 | -5.9 |
| telmisartan | -4.6 | -3.7 | -2.0 | -2.0 | -3.9 |
| dabigatran | -4.8 | 0.7 | 11.1 | 11.1 | 0.0 |
| valsartan | -4.9 | -3.1 | 0.0 | 0.0 | -3.5 |
| gemfibrozil | -4.9 | -3.0 | 0.5 | 0.5 | -3.3 |
| enoxaparin | -4.9 | -8.0 | -13.5 | -13.5 | -6.9 |
| mycophenolate | -4.9 | -8.1 | -13.7 | -13.7 | -7.1 |
| lisinopril | -5.0 | -3.5 | -0.8 | -0.8 | -3.8 |
| terazosin | -5.1 | -2.9 | 1.3 | 1.3 | -3.4 |
| captopril | -5.2 | -4.2 | -2.4 | -2.4 | -4.4 |
| irbesartan | -5.3 | -3.5 | -0.3 | -0.3 | -3.8 |
| ramipril | -5.5 | -2.5 | 3.1 | 3.1 | -3.1 |
| doxazosin | -5.6 | -3.5 | 0.3 | 0.3 | -4.0 |
| valganciclovir | -5.7 | -12.9 | -25.7 | -25.7 | -10.5 |
| atenolol | -5.8 | -1.7 | 5.8 | 5.8 | -2.6 |
| repaglinide | -5.9 | -4.0 | -0.9 | -0.9 | -5.0 |
| levetiracetam | -5.9 | -3.4 | 1.5 | 1.5 | -3.8 |
| enalapril | -6.1 | -5.0 | -3.0 | -3.0 | -5.2 |
| benazepril | -6.5 | -5.1 | -2.5 | -2.5 | -5.4 |
| febuxostat | -6.6 | -4.2 | 0.3 | 0.3 | -4.7 |
| buprenorphine | -6.8 | -3.8 | 1.6 | 1.6 | -4.5 |
| nadolol | -6.9 | 0.9 | 15.3 | 15.3 | -0.7 |
| lacosamide | -7.2 | -5.9 | -3.4 | -3.4 | -6.0 |
| exemestane | -7.2 | -5.4 | -2.4 | -2.4 | -6.1 |
| eplerenone | -8.4 | -5.8 | -1.1 | -1.1 | -6.3 |
| alirocumab | -9.0 | -4.5 | 4.0 | 4.0 | -5.1 |
| testosterone | -9.4 | -10.4 | -12.0 | -12.0 | -10.1 |
| propafenone | -9.5 | -6.0 | 0.7 | 0.7 | -6.4 |
| vilazodone | -10.9 | -11.4 | -11.9 | -11.9 | -10.7 |
| bisoprolol | -11.1 | -4.3 | 8.3 | 8.3 | -5.7 |
| sacubitril | -11.3 | -5.7 | 4.4 | 4.4 | -7.3 |
| digoxin | -21.0 | -17.5 | -11.0 | -11.0 | -18.3 |
