# Supplementary material for: High-Throughput Assessment of Real-World Medication Effects on QT Interval Prolongation: Observational Study
Source: JMIR Cardio. 2023 Jan 20;7:e41055. doi: 10.2196/41055 (PMC9898836; doi:10.2196/41055)
Supplement: Multimedia Appendix 2 [file cardio_v7i1e41055_app2.docx]

*Supplemental Table 2. Change in heart rate when comparing ECGs on versus off of medication.*

| **Med** | **Change in Heart Rate (beats per minute) (95% CI)** |
| --- | --- |
| amiodarone | -4.8 (-5.3, -4.3) |
| disopyramide | -7.9 (-10.4, -5.2) |
| dofetilide | -1.6 (-7.1, 4.2) |
| fluoxetine | -2.5 (-3.1, -1.9) |
| lactulose | -0.9 (-1.6, -0.2) |
| lenalidomide | -2.1 (-4, -0.4) |
| methadone | -1.6 (-3, -0.3) |
| metolazone | -0.8 (-1.8, 0.2) |
| mexiletine | -6.5 (-9.5, -3.5) |
| midodrine | -0.7 (-1.6, 0.2) |
| rifaximin | 1 (0, 1.9) |
| sotalol | -3.5 (-4.8, -2.1) |
| verapamil | -1.3 (-2.3, -0.3) |
| citalopram | -1.5 (-2.1, -0.9) |
| mercaptopurine | -5.4 (-7.6, -3.2) |
| ritonavir | -2.5 (-4.2, -1) |
| anastrozole | 0.7 (-0.2, 1.7) |
| cinacalcet | -0.5 (-1.2, 0.2) |
| darunavir | -3.7 (-5.4, -2.2) |
| escitalopram | -2.3 (-2.7, -1.9) |
| furosemide | -0.5 (-0.7, -0.2) |
| lithium | -3.1 (-4.8, -1.5) |
| methotrexate | -0.8 (-1.9, 0.2) |
| ranolazine | -0.2 (-1, 0.7) |
