# Supplementary material for: High-Throughput Assessment of Real-World Medication Effects on QT Interval Prolongation: Observational Study
Source: JMIR Cardio. 2023 Jan 20;7:e41055. doi: 10.2196/41055 (PMC9898836; doi:10.2196/41055)
Supplement: Multimedia Appendix 3 [file cardio_v7i1e41055_app3.docx]

*Supplemental Table 3. Changes in QTc when on a medication compared to when off a medication before or after being on the medication.*

|  | ECGs Off Med Before Being on Med | | ECGs Off Med After Being on Med | |
| --- | --- | --- | --- | --- |
| **Medication** | **N** | **QTc change (95% CI)** | **N** | **QTc Change (95% CI)** |
| amiodarone | 3064 | 16 (14.5, 17.4) | 855 | 11.4 (9.3, 13.7) |
| disopyramide | 117 | 10.1 (2.5, 17.6) | 53 | -6.3 (-17.1, 4.5) |
| dofetilide | 28 | 26.4 (13.7, 39) | 10 | 7.1 (-4.8, 18.9) |
| fluoxetine | 1243 | 4.6 (2.9, 6.3) | 820 | 5.3 (3.3, 7.3) |
| lactulose | 2854 | 8.8 (7, 10.7) | 1305 | 7.6 (5.4, 9.9) |
| lenalidomide | 283 | 8.8 (4.6, 12.9) | 153 | 1 (-4.1, 6.5) |
| methadone | 614 | 7.6 (4.1, 11.2) | 294 | 3 (-0.9, 6.9) |
| metolazone | 969 | 7.9 (5, 11) | 619 | 12.6 (9.7, 15.8) |
| mexiletine | 71 | 19.4 (7.7, 30.7) | 45 | 16.8 (4.9, 29.7) |
| midodrine | 1422 | 7.3 (4.7, 10.1) | 369 | 5.7 (2.2, 9) |
| rifaximin | 1480 | 13.6 (11, 16.1) | 714 | 16.2 (13.2, 19) |
| sotalol | 322 | 13.3 (9.2, 17.1) | 161 | 4.9 (-0.8, 10.4) |
| verapamil | 429 | 8.5 (5.5, 11.4) | 337 | 3.3 (0, 6.4) |
| citalopram | 1176 | 5.7 (3.9, 7.5) | 922 | 3.7 (1.7, 5.7) |
| mercaptopurine | 94 | 3.6 (-3, 9.4) | 60 | 0 (-6.3, 6.7) |
| ritonavir | 147 | 0.4 (-4.6, 5.8) | 143 | 7.2 (2, 12.6) |
| anastrozole | 756 | 5.9 (3.5, 8.5) | 259 | 4.6 (1, 8.2) |
| cinacalcet | 1105 | 7.4 (5.3, 9.6) | 385 | 2.7 (-0.2, 5.5) |
| darunavir | 157 | 3.4 (-1.8, 7.7) | 83 | -0.8 (-7.1, 5.5) |
| escitalopram | 4353 | 3.3 (2.2, 4.3) | 1989 | 1.6 (0.3, 2.9) |
| furosemide | 8559 | 6.8 (6.1, 7.6) | 3572 | 3.8 (2.8, 4.8) |
| lithium | 230 | 0.3 (-3.6, 4.5) | 200 | 4.1 (0, 7.9) |
| methotrexate | 470 | 4.8 (2, 7.6) | 368 | 4.6 (1.7, 8.1) |
| ranolazine | 929 | 9.1 (7, 11.2) | 269 | 5.2 (1.9, 8.5) |
